# Caesarean Scar Choriocarcinoma: Ultrasound and Magnetic Resonance Imaging Findings

**DOI:** 10.5334/jbr-btr.950

**Published:** 2016-03-07

**Authors:** Tumay Bekci, Mesut Ozturk, Murat Danaci

**Affiliations:** 1 Ondokuz Mayis University Faculty of Medicine, TR

**Keywords:** Caesarean scar, choriocarcinoma, ultrasound, magnetic resonance imaging, uterus

## Abstract

Primary gestational choriocarcinoma in a uterine caesarean section scar (CSS) is an extremely rare entity, and its timely diagnosis and treatment is crucial in order to prevent related complications and metastatic disease. Herein, we report on a 33-year-old female who was referred to our department with an initial diagnosis of ectopic pregnancy. Transabdominal ultrasound and magnetic resonance imaging (MRI) demonstrated a nodular mass on CSS. The final histopathological diagnosis was CSS choriocarcinoma.

## Introduction

Choriocarcinoma is a rare form of gestational trophoblastic disease (GTD), and its most common site of origin is the uterus. It may be associated with ectopic pregnancies, but these cases are extremely rare. There is only one case report in the literature presenting the transvaginal ultrasound findings of a caesarean section scar (CSS) choriocarcinoma [1]. Herein, we present a unique case of a CSS choriocarcinoma, with transabdominal ultrasound and magnetic resonance imaging (MRI) findings. To the best of our knowledge, transabdominal ultrasound and MRI findings of a CSS choriocarcinoma are reported for the first time in the literature.

## Case Report

A 33-year-old female, gravida 2 para 1, was referred to our university hospital from a local medical clinic with a history of a positive serum beta-Human chorionic gonadotropin (β-hCG) level but no visible intrauterine gestational sac. Her laboratory findings were all normal except for the serum β-hCG, which was 146.762 mIU/mL. A gynecological examination revealed a closed cervical os and mild adnexal tenderness. The patient had regular 28-day menstrual cycles. Her first pregnancy had ended with a full-term delivery by caesarean section approximately nine years earlier. There was no history of cancer in her family.

**Figure 1:**
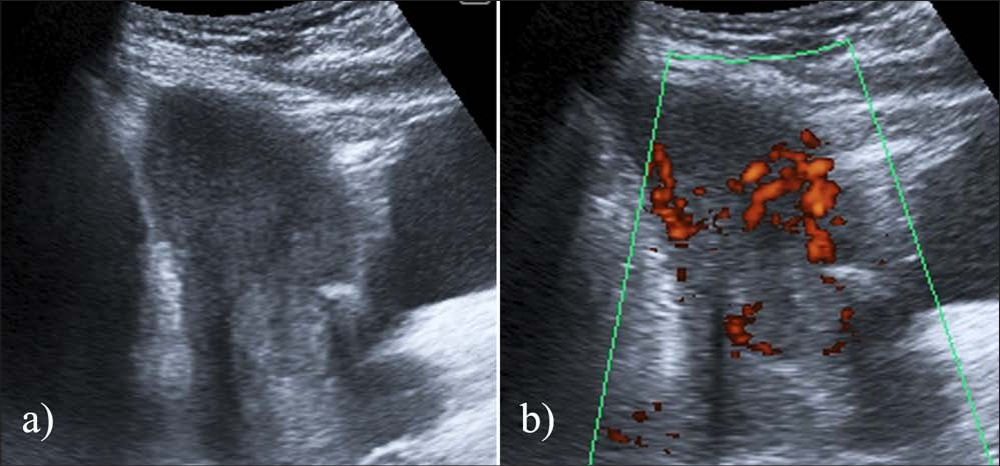
Transabdominal grayscale (a) and Doppler ultrasound (b) images show the lesion in the anterior inferior wall of the uterus. It is an echogenic, peripherally vascular soft-tissue mass with no sac-like structure or embryo.

A grayscale transabdominal ultrasound showed an empty uterine cavity, but a round hyperechoic lesion was identified in the anterior inferior wall of the uterus, measuring 23x32 mm in diameter (Figure 1). There was no visible embryo or yolk sac within the lesion. The patient underwent careful dilatation and curettage (D&C) procedure, and the histopathological results were consistent with an ectopic molar pregnancy. The post-D&C period was uneventful, without excessive bleeding. The patient scheduled for methotrexate therapy, and the patient’s serum β-hCG level showed significant decrease.

**Figure 2:**
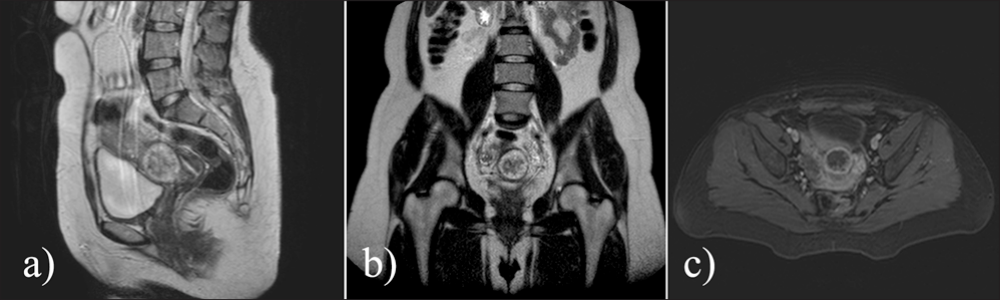
T2-weighted sagittal (a) and coronal (b) MR images demonstrate a peripherally hyperintense and centrally hypointense lesion located in the cesarean section scar of the uterus. The lesion demonstrated peripheral contrast-enhancement after gadolinium administration (c).

The β-hCG level that became available to us on the twenty-third day of the treatment was still as high as 15.947 mIU/mL, and it then rose again. Therefore, the patient underwent an magnetic resonance imaging (MRI) examination, which demonstrated a peripherally hyperintense and centrally hypointense lesion on coronal and sagittal T2-weighted images of the uterine CSS. After gadolinium administration, the lesion demonstrated peripheral contrast enhancement on T1-weighted fat-suppressed axial images (Figure 2). The differential diagnosis included a malignant GTD, and surgery was planned. Selective uterine artery embolization was performed immediately before the surgery to reduce the risk of intraoperative bleeding, and a total abdominal hysterectomy was then performed. The patient’s postsurgical recovery was uneventful and her serum β-hCG level decreased. The histopathological examination confirmed a friable hemorrhagic mass in the anterior inferior wall of the uterus, adherent to the previous CSS. It was composed of atypical cytotrophoblastic cells with high mitotic activity and myometrial invasion, and intensive necrosis and hemorrhage were present centrally. Immunocytochemical staining for β-hCG was positive and the Ki-67 index was as high as 80%. The final pathologic diagnosis was choriocarcinoma.

A metastatic workup revealed multiple lung metastases. There was no evidence of metastasis to the brain. Adjuvant chemotherapy was planned and the patient received four cycles of the EMA/CO regimen (etoposide, methotrexate, actinomycin D, cyclophosphamide, and vincristine). She tolerated the therapy well, and weekly serum β-hCG measurements showed a significant drop, from 12.459 mIU/ml after the surgery to 0.7 mIU/ml following chemotherapy. A follow-up CT scan showed a significant reduction in the size of the lung metastases.

## Discussion

Gestational trophoblastic disease (GTD) is the abnormal proliferation of pregnancy-associated trophoblastic tissue with malignant potential [2]. It includes the tumor spectrum of hydatidiform mole, invasive mole, choriocarcinoma, and placental-site trophoblastic tumors. Choriocarcinoma is a malignant histological subtype of GTD that may follow a normal-term pregnancy, an abortion, an ectopic pregnancy, or a hydatidiform mole [1]. Most of the time, it arises in the uterine body, but extrauterine locations such as ovary [3], fallopian tube [4], vagina [5], vulva [6], gut [7], and CSS [1] have also been also reported. A CSS pregnancy is an ectopic pregnancy in which the gestational sac is seen within the myometrium of a previous cesarean scar. Its incidence is 1:2,216, with a rate of 6.1% in women with ectopic pregnancies and at least one previous cesarean section [8]. Choriocarcinoma in a CSS is an extremely rare entity; there is only one case report found in the literature [1]. In the present case report, we present a CSS choriocarcinoma with transabdominal ultrasound and MRI findings. To the best of our knowledge, these imaging features are presented for the first time in the literature.

Although it is extremely rare, choriocarcinoma must be considered in the differential diagnosis of ectopic pregnancies within cesarean scars. An early diagnosis requires high suspicion, and it is crucial to reduce the potential complications. It is difficult to predict the prognosis of choriocarcinoma in CSS, as there are very few reported cases, but when we consider the aggressiveness of this type of lesion, life-threatening complications such as uncontrollable hemorrhage and uterine rupture can be presumed. Therefore, it is crucial to make a prompt and accurate diagnosis.

Hydatidiform mole has the sonographic appearance of a solid collection of echoes with numerous anechoic spaces [2]. When the volume of the mole is too small, it may be perceived as an echogenic mass filling the uterine cavity, and MRI may be performed to determine whether the molar tissue extends outside the uterus. On T2-weighted MR images, the hydatidiform mole appears as a heterogeneous mass of high signal intensity that distends the endometrial cavity. Numerous cystic spaces may be present in the mass [2]. The differential diagnosis of a hydatidiform mole or choriocarcinoma is very difficult without a pathological examination. Consistent or rising gonadotropin levels in the absence of pregnancy or following evacuation of a pregnancy, or in the presence of metastatic lesions in a patient with a known molar pregnancy may help to establish the diagnosis of choriocarcinoma. Radiological findings are indistinguishable except in cases in which obvious extension into the parametrium can be visualized. MR imaging may be able to demonstrate soft-tissue extension, so it may contribute more to the differentiation of these two entities.

In conclusion, CSS choriocarcinoma is an extremely rare entity, and imaging and a timely diagnosis are the keys to its successful treatment and the prevention of complications. By presenting a choriocarcinoma case within the CSS, we aimed to draw the attention of both radiologists and clinicians, who should consider this rare entity in the differential diagnosis of cesarean scar pregnancies.

## Competing Interests

The authors declare that they have no competing interests.
